# Integration of Digital Twin, Machine-Learning and Industry 4.0 Tools for Anomaly Detection: An Application to a Food Plant

**DOI:** 10.3390/s22114143

**Published:** 2022-05-30

**Authors:** Giovanni Paolo Tancredi, Giuseppe Vignali, Eleonora Bottani

**Affiliations:** Department of Engineering and Architecture, University of Parma, 43124 Parma, Italy; giovannipaolo.tancredi@unipr.it (G.P.T.); giuseppe.vignali@unipr.it (G.V.)

**Keywords:** digital twin, machine learning, food plants, Industry 4.0, monitoring, safety

## Abstract

This work describes a structured solution that integrates digital twin models, machine-learning algorithms, and Industry 4.0 technologies (Internet of Things in particular) with the ultimate aim of detecting the presence of anomalies in the functioning of industrial systems. The proposed solution has been designed to be suitable for implementation in industrial plants not directly designed for Industry 4.0 applications. More precisely, this manuscript delineates an approach for implementing three machine-learning algorithms into a digital twin environment and then applying them to a real plant. This paper is based on two previous studies in which the digital twin environment was first developed for the industrial plant under investigation, and then used for monitoring selected plant parameters. Findings from the previous studies are exploited in this work and advanced by implementing and testing the machine-learning algorithms. The results show that two out of the three machine-learning algorithms are effective enough in predicting anomalies, thus suggesting their implementation for enhancing the safety of employees working at industrial plants.

## 1. Introduction

A current challenge related to industrial systems is transferring manufacturing processes to the Internet of Things (IoT) or cyber–physical systems (CPS), which means that objects and their virtual representation should be networked with others [1]. The IoT is a network of physical objects embedded with sensors, software, and other technologies for the purpose of connecting and exchanging data with other devices and systems over the internet; it thus collects and shares data between smart devices to carry out data monitoring and control in cyber–physical systems (CPS). Artificial intelligence (AI) solutions applied to CPSs allow for machine learning (ML) inference on numerous data that can be acquired with ever-increasing accuracy, thanks to the possibility of training ML models with massive amounts of information generated by IoT devices [2]. With the introduction of ML techniques in the Industry 4.0 paradigm, each industrial field can take advantage of real-time monitoring and control of the production processes, thanks to the effective data analysis and prediction enabled by these techniques. Combined with the development of IoT and data analysis technologies, CPS has massively increased the performance of industrial processes [3].

The development of a digital environment, commonly known as a digital twin (DT) and envisioned as a replica of a physical system, is a valuable supporting technique for predicting system parameters and checking the effectiveness of a ML model. A DT describes a system in analytic form, with the aim of mirroring the effective status of its physical counterpart. CPS integration, data sharing, and system communication in a DT involve a series of tasks that have to mediate hardware reliability, model robustness, and secure, real-time data communications with low latency. Nevertheless, the hardware requirements, software developed, network infrastructure, communication protocols, and the data analysis chosen can affect the concrete usability of the developed DT.

In turn, ML techniques in industry have been applied successfully in various sectors, such as predictive maintenance, quality management, and zero-defect manufacturing [4]. In the context of predictive maintenance, IoT sensors are typically used for collecting raw data that allow to monitor different operating parameters (such as temperature, pressure, product flow, etc.) of industrial machines during their functioning. These raw data can then be processed and analyzed using ML tools that, thanks to the training previously received, are typically able to discover an underlying relationship between the different parameters of the system. On the basis of said relationships and on the output predicted by the ML model, a decision maker can, for instance, understand if a part of the equipment must be replaced or forecast its probability of failure in the medium term [5]. As the amount of data and case studies increase, ML techniques can improve their performance and provide more accurate predictions. This process can radically change the traditional way of organizing industrial maintenance; indeed, maintenance activities have been typically organized as interventions at regular time intervals, often without the opportunity of knowing the real conditions of the machines in advance. Such an approach, however, does not prevent the possibility of sudden failures of the plant, which in turn would lead to unplanned interventions and consequent production downtime. Besides predictive maintenance, ML systems can also be used in quality control to detect the possible defects of a final product, with a margin of error close to zero [6]. ML has also been widely used in the field of anomaly detection [7] due to its ease of use, and in the field of fault detection [8]. In the field of predictive maintenance, Çınar et al. [9] reviewed the ML approaches suitable for implementation when trying to minimize downtime and maximize the utilization rate and the useful life of equipment.

Although DT, IoT, and ML are all quite popular in literature, some research gaps remain for their practical application, and need to be addressed. First, the combined usage of DT, IoT, and ML is quite unexplored. Only a few architectures have been developed to integrate DT and ML techniques. For instance, neural networks (NN) have been integrated into a DT environment to evaluate surface roughness of a mill, using real-time data acquisition [10]. Similarly, a DT driven by ML algorithms has been proposed by [11] to be implemented in a petrochemical factory. These few studies have proposed an integrated software and hardware solution grounded on information and digital data exchange. As a second point, looking at real case applications, in the food industry there are just a few examples of practical implementation of ML algorithms. The multiple linear regression algorithm and support vector machine have been recently implemented in a dry system [12], to predict the drying kinetics of a solar drier, or to perform quality control on fruits aided by NN [13]. Once again, however, none of the analyzed studies has proposed a ML-driven DT model. On the contrary, in food plants, traditional control systems are typically used [14], while DT models, which are more user friendly, are rarely available [15]. For the same reason, applications of these tools for predicting machine anomalies in food plants also are lacking in the literature.

Based on these premises and on the gaps highlighted, this work aims to describe a straightforward solution for anomaly detection, suitable to be adopted in industrial plants not directly designed for Industry 4.0 applications. To be more precise, the basic idea of the manuscript is to delineate an approach for implementing various ML tools into a DT environment and applying them to a real plant. As such, this paper starts from two previous studies; in the first one, the DT environment was developed for the industrial plant under investigation [16], while the second one discussed the results obtained with the DT for monitoring some selected plant parameters [17]. This paper integrates the previous findings into an online tool, and at the same time, introduces the advancements achieved compared to the previous studies, thanks to the implementation of various ML algorithms. Indeed, because of the relatively small number of studies that have applied ML techniques to the area of anomaly detection, this study is exploratory in nature, and aims to evaluate the performance of different algorithms and search for the best solution for anomaly detection in a real plant. In line with this aim, three ML models were selected for implementation, choosing tools representative of various ML categories. In particular, the selected ML models reflect a supervised regressor (i.e., a multiple linear regression), a supervised classifier (a multi-layer perceptron), and an unsupervised algorithm (the k-means clustering).

The remainder of the paper is organized as follows. Section 2 describes the materials and methods used in carrying out the research; these include the pilot plant, the procedure followed for data collection, and the ML algorithms developed. Section 3 describes the testing procedure followed for evaluating the effectiveness of the proposed ML algorithms, both using the sample data and online data from the plant. Conclusions and future research directions are discussed in Section 4.

## 2. Materials and Methods

### 2.1. Pilot Plant

The system under examination is a pilot plant that consists of a pasteurization system equipped with a counter-flow tube-in-tube heat exchanger used for pre-heating fluid foods (process fluids) via the transfer of heat by water (service fluid), in turn, pre-heated with steam provided by a steam generator. This latter part is able to provide heated steam at up to 11 bar, while the pasteurization system can process up to 5 m^3^/h of product. For this plant, a DT model was developed and tested in previous studies [16,18].

A set of probes mounted on the pasteurization system provide an analog signal via a current loop of 4–20 mA, useful for describing the machine status by monitoring the product flow and the pressure at the inlet and outlet of the heat exchanger. Due to the low pressure generated by the fluid, some precision probes have been installed on the plant (model S11 manufactured by Wika, Klingenberg, Germany; www.wika.com, accessed on 1 March 2022); these probes are designed for being used with viscous fluids, which would occlude the probe channel. Table 1 reports the main characteristics and models of the sensors installed. All the sensors have a response time of less than 2 ms, a high accuracy evaluated in 0.5% of the span (i.e., the difference between the lowest and the actual output signal), and a non-linearity lower than 0.2% of the best-fit straight line.

For measuring the flow of products with possible pieces, a mass flowmeter (model Optimass manufactured by Krohne, Duisburg, Germany; https://krohne.com/en, accessed on 1 March 2022) was installed after a twin-screw pump (manufactured by Bornemann, Obernkirchen, Germany, https://www.bornemann.com, accessed on 1 March 2022). The chosen sensor has an accuracy of ±0.15% of the measured flow rate.

### 2.2. System Architecture

In line with the aim of this study, as previously mentioned, this paper proposes the integration of ML tools into a DT environment, which models the functioning of the system detailed above.

The DT model developed consists of:

- A physical layer, which includes the pilot plant, the hardware for data acquisition and signal generation, and a wireless adapter.
- A digital layer developed using LabVIEW software and Python language. LabVIEW, in turn, makes use of two software development environments. The first one, called “front panel”, reflects the human–machine interface (HMI) in which the user can monitor the system, change the input parameters, and generate the output signals. The second, called “block diagram”, is the background of the software; it is coded in G-language and is embodied in the so-called virtual instruments.

**Figure 1:**
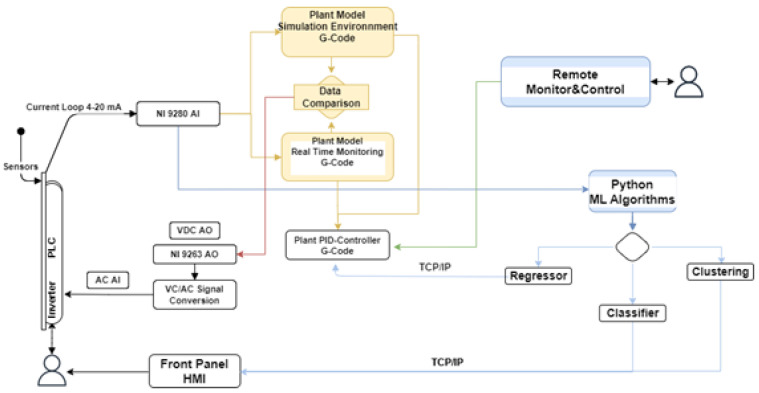
System architecture.

The overall architecture of the solution developed, highlighting the integration of ML and DT, is proposed in Figure 1.

As can be seen from Figure 1, the system can work in four different modes. The first scenario involves the usage of the system as a “Plant model simulation environment”. This basically means that the equations implemented in DT model are used to reproduce the physical properties of a product as a function of its flow and temperature, and evaluate, as output, the heat exchanger pressure drop on the basis of the geometry of the pasteurization system and the rheological properties of the fluid. Using this tool, the user can simulate the machine status by varying the process parameters, and if needed, trigger an analog signal by adjusting the analog voltage output or the proportional–integral–derivative (PID) controller setpoint. The second functionality refers to a fully automated condition called “Real-time monitoring”, in which the signals are directly acquired from the plant sensors. On the basis of the signals acquired and on the manual adjustment of the PID setpoint, the digital environment returns an analog output to drive the motor pump and control the product flow. Then, using the “Data comparison” tool, the system can trigger an analog output signal by comparing the pressure drop analytically computed via DT with the one evaluated by the signal acquired, and adjusts the product flow according to the current machine status. The third setting, labeled, “Remote monitor and control”, allows for controlling the machine status and generating a voltage output via remote connection to the digital environment. Finally, the fourth mode assumes an ML-based algorithm is embodied in the system and developed to drive the motor pump autonomously, or to display a message on the front panel, i.e., the HMI of the pilot plant. This environment, which will be referred to as “ML algorithm”, has been built with the combined usage of Python and G-Code (see Figure 1). In an attempt to evaluate various solutions, their performance, and their capability for providing useful information to the user, three ML models have been implemented, namely, a linear regressor, a classifier, and a clustering algorithm.

**Figure 2:**
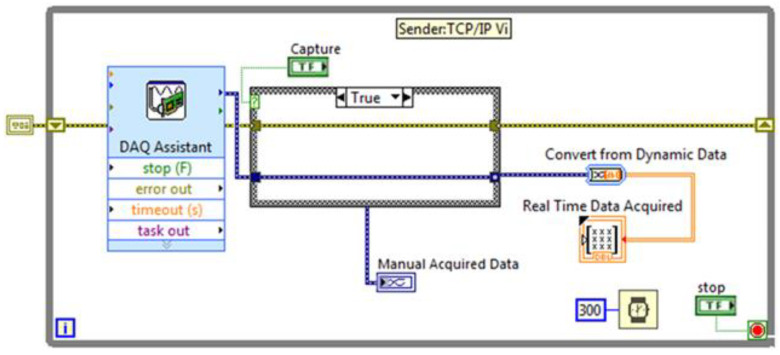
Scheme of the TCP/IP sender.

**Figure 3:**
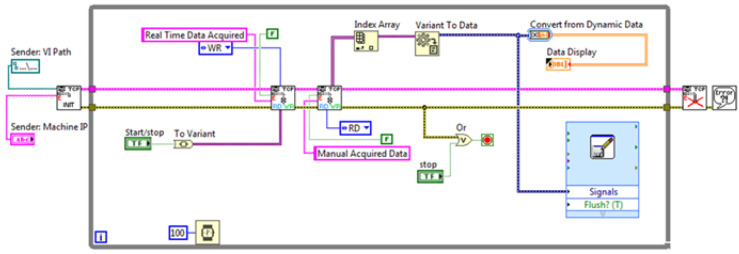
Scheme of the TCP/IP receiver.

Among the variety of communication protocols that have been suggested as standard for data sharing with the host involved in the DT environments, the TCP/IP (Transmission Control Protocol/Internet Protocol) suite was chosen for implementation. This protocol provides syntactic and semantic rules for communication, contains the details of the message formats, and describes how a computer responds when a message arrives and how it handles errors or other abnormal conditions. In addition, it allows for describing the communication between various computers independently on the network hardware. In the case under examination, the protocol adopted allows for data communication between the NI-9208 and a host computer that can perform the data analysis without affecting the computational complexity of the embedded system. To this end, two G-Code programs have been developed. The first program for data sending is installed on the data acquisition module, while the second one, i.e., the TCP/IP receiver, has been embodied as a sub-VI in the block diagram of the digital layer (Figure 2 and Figure 3).

This TCP/IP infrastructure has been built with the specific aim of enabling real-time data acquisition from the plant, its storage on a client host, and the possibility of forecasting the output on the basis of the real-time input acquired and the elaboration made by the ML algorithms. The forecast will be obtained using the ML algorithms implemented in the solution; depending on the chosen algorithm and on the elaborations made, this layer can provide two different outputs.

To be more precise, the classification and the clustering algorithms return a message box in which the machine status is displayed. In particular, the evaluation of the inlet pressure has been carried out using the data comparison module of the DT [18] by inserting the rheological properties of each fluid (taken from [19]) and analytically computing the pressure drop of the pasteurization system at different flow rates. On the basis of the combined evaluation of the flow rate (F), inlet pressure (P1), and outlet pressure (P2), the system status can fall into one of the following categories:

- *Ok*: The parameters are in the correct range of values and therefore the machine is working correctly. Based on the result obtained via the DT model, the “correct” functioning is assumed to be described by values that deviate between 0 and 10% (in absolute terms) from the value computed by the DT;
- *Warning*: One or more parameters are out of the correct range of functioning as previously defined, and in particular, the numerical value of the parameter deviates by 10–25% (in absolute terms) from the value computed by the DT. Under this circumstance, possible working anomalies could arise. This status could also describe a transitory phase, in which the plant moves from an original steady-state condition to a subsequent one, and therefore some parameters deviate from the normal values;
- *Failure*: The machine is experiencing a working problem and its functioning needs to be stopped. This circumstance occurs when the absolute deviation between the real and computed values is greater than 25%.

The range for the values of the three categories above has been defined on the basis of previous studies carried out by the authors on the plant under examination [16,17,18,19], as well as exploiting the developed knowledge about the plant functioning. It is worth mentioning that, although a classification of the various statuses of machine functioning has been proposed by various authors in literature, to the best of the authors’ knowledge there are no recognized criteria for defining those statuses in a rigorous way. Nonetheless, the ranges used in this study are similar to those found in previously published studies (cf., e.g., [20]). Moreover, for enhancing the possibility of detecting the plant malfunctioning (i.e., the failure condition) against the warning and ok statuses (which are obviously less problematic), a wider range of possible values has been assigned to the former category (75% of the cases) compared to the remaining two categories.

The corresponding information, i.e., the status detected, will be displayed on the front panel HMI via TCP/IP receiver G-Code on the server.

The linear regression model, instead, returns a discrete value, which reflects the estimate of the (selected) output variable (i.e., the outlet pressure in this study); this value can be sent to the plant PID controller to adjust the setpoint based on the current machine status.

### 2.3. Data Collection

For building the dataset used for applying the ML algorithms, a series of machine tests were carried out using three different fluids. To be more precise, the machine was tested with water (fluid 1), and then, to emulate the behavior of two non-Newtonian food fluids, we used two mixtures of water and a food additive as a gelling agent (i.e., Gellan Gum) in different percentages. Fluid 2 had a Gellan Gum percentage of 0.1% while fluid 3 had a 0.15% mass composition (*w*/*w*). The signals returned by each sensor were acquired with a sampling rate of one sample per second. The testing phase was run by varying the process flow acting on an inverter frequency with a 10% step (Table 2) and setting three different operating temperatures, owing to the dependency of the rheological properties of the additive on temperature [19].

For collecting data relating to the various conditions of functioning (i.e., ok, warning, or failure), the machine was stressed by varying one process parameter at a time and maintaining the remaining parameters at a steady state. To achieve a variation in the inlet pressure in a range between 0 and 185 mbar, without varying the outlet pressure or the product flow, manual actions were made on the inlet valve mounted immediately next to the product pump. Such actions involve increasing the inlet pressure due to the pipe section reduction, without simultaneously affecting the product flow and outlet pressure. To vary the outlet pressure, the outlet section of the heat exchanger was gradually closed by acting on the manual valve mounted at the outlet of the heat exchanger. To avoid unsafe conditions for the operator or corrupted machine functioning, the outlet valve was opened once the outlet pressure achieved the maximum measurable value of 250 mbar. Finally, the product flow was varied by gradually closing the inlet product valve mounted at the outlet section of the product tank.

**Figure 4:**
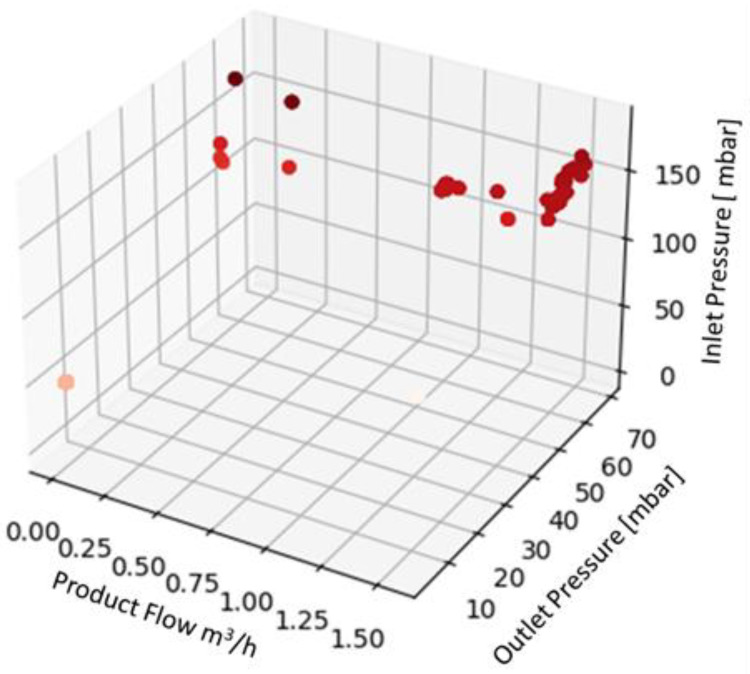
Three-dimensional plot of the dataset for water.

**Figure 5:**
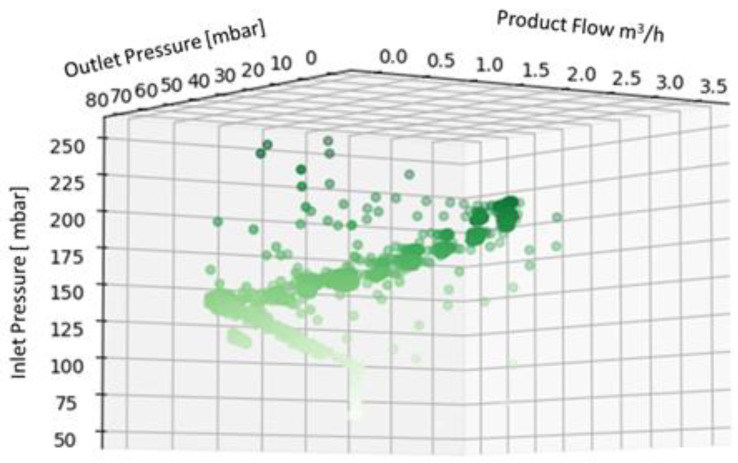
Three-dimensional plot of the dataset for Fluid 2.

**Figure 6:**
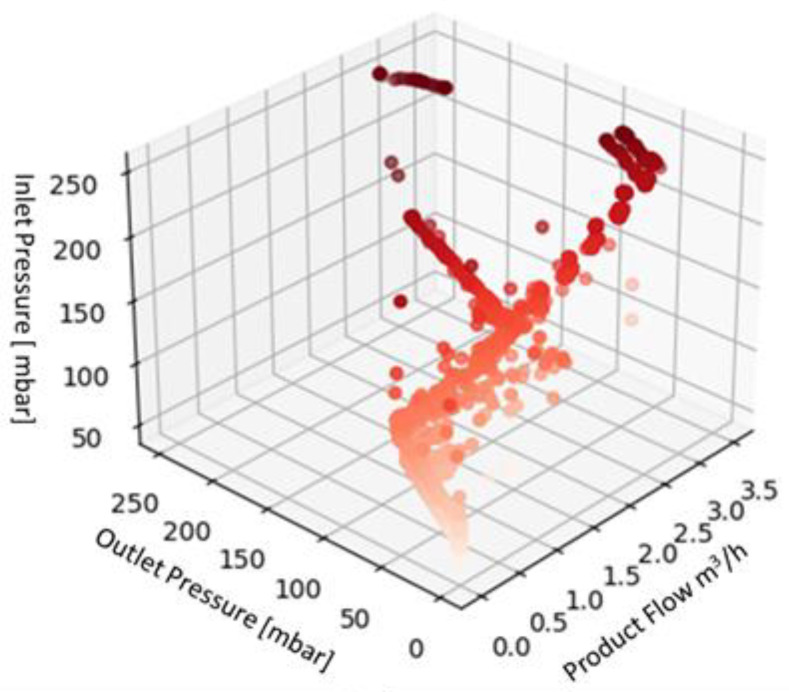
Three-dimensional plot of the dataset for Fluid 3.

The data acquired during the machine test were collected in a dedicated database for each fluid and preprocessed before using them as input for the ML algorithms. The preprocessing phase was carried out following the steps listed below:

- Checking each triplet of collected data (flow, inlet pressure, and outlet pressure);
- Adding a new column, named “label”, to each triplet of values collected. The label was used to describe the machine status on the basis of the values of the considered parameters and knowing the testing scenario. For a triplet of values describing a normal condition, the label assigned is “ok”, while for values collected during a transitory phase, the label assigned is “warning”. Anomalous situations, according to the description provided above, were labeled as “failure”;
- Describing the data collected by evaluating their average, standard deviation, maximum and minimum read values, and percentiles for the first quartile (25%) and third quartile (75%). These values are summarized in Table 3 for the three fluids, in order to provide the reader with an overview of the data collected. The whole dataset is available in the Supplementary Materials of this paper;
- Displaying the data in 3D plot using Python in order to provide a preliminary overview of the distribution of the data collected (Figure 4, Figure 5 and Figure 6), aided by colors’ graduation which highlights the density of data in the 3D graph’ space.

Overall, the dataset resulting from preprocessing consists of 6256 rows and 4 columns for each fluid. The four columns reflect the machine status and the process parameters considered; in particular, the first and the second list the inlet pressure and the outlet pressure of the process fluid in mbar, as they were measured at the inlet and outlet sections of the heat exchanger. The third column lists instead the values of the product flow in m^3^/h. The data were saved in the form of a .csv file in Microsoft Excel^TM^ release 2016 for Windows (Microsoft Corporation, Redmond, WA, USA).

### 2.4. Machine-Learning Algorithms

The selected ML algorithms were programmed and implemented using the scikit-learn package (https://scikit-learn.org/stable/; accessed on 18 January 2022) of Python as the development environment.

The script of the supervised ML algorithms, namely, multiple linear regression and artificial neural network, follows the same structure and consists of three sections. In the first section, the data are imported from the dataset of values collected during the tests. Some preliminary elaborations are also made on the set of data to make them suitable for processing by the algorithms. As an example, the data format was converted into the appropriate type (object-type data), while the process parameters variables were converted into floating-type data. A second section of the script is used for splitting the dataset into two subsets, namely, the training and the testing sets, accounting for 70% and 30% of the original set of data, respectively. Then, the ML model is trained on the first sub-set of data. Once the algorithm has been trained, the last section of the script carries out the model prediction. A cross-validation step was carried out, by varying the composition of the sets used for training and testing (while keeping their percentages unchanged), to check whether the results of the supervised algorithms could be somehow dependent on the specific elements belonging to these sets. The result was negative, meaning that the algorithm performance does not depend on the selected training and testing sets.

The third algorithm developed is instead an unsupervised clustering model. This kind of model does not require an output on which to be trained; rather, the training and testing phases are not carried out at all. For the purpose of our application, the expected outcome of this algorithm is the classification of the triplets of data collected into the appropriate status of “ok”, “warning”, or “failure”. Due to the lack of a testing phase, the only way to check whether the result of the algorithm can be considered as correct is to compare the clusters identified for the triplets of data with the real status of the machine as it was preliminary set in the database. This means that, ideally, the algorithm should group the triplets of the dataset into 3 clusters, corresponding to the “ok”, “warning”, or “failure” status. The possible advantage of an unsupervised algorithm, therefore, is that, in the case of exact classification of the clustered data, in future applications there would be no need to preliminarily classify the data into the “ok”, “warning”, or “failure” categories.

## 3. Testing Methodology

### 3.1. Multiple Linear Regression

Multiple linear regression is a linear approach for modeling the relationship between a response, called the dependent variable, and more than one explanatory variable, called the independent variables [21]. In the case under examination, this model makes use of two independent variables, referred to as *x*_1_ and *x*_2_, which reflect the outlet pressure and product flow, respectively; as the dependent variable, for which the prediction should be made, the inlet pressure is chosen (*y*). The general linear model describing the dependent variable as a function of the independent variables is shown in Equation (1):

*y* = *a*_1_*x*_1_ + *a*_2_*x*_2_ + *intercept* (1)

In Equation (1), *a*_1_ and *a*_2_ represent the regression coefficients, while *intercept* is a fixed value. These three parameters were estimated using the dataset described in Section 2.3; related results for the three fluids under examination are listed in Table 4.

As metrics for estimating the accuracy of the models, the mean absolute error (MAE), and the coefficient of determination (R^2^) were computed. The former is a measure of the error between paired observations expressing the same phenomenon [22]; in the case under examination, the MAE thus reflects the mean of the absolute error computed by comparing the real values of the dataset with those predicted by the linear regression model. The R^2^ denotes instead the proportion of the variation in the dependent variable that is predictable from the independent variables [23]; it thus measures how the predicted values fit with the real data. The maximum fitting score can be achieved at R^2^ equal to 1.

The outcomes in Table 4 clearly show that, for the case under examination, R^2^ validates the ML algorithm developed, since the results obtained for each fluid reach R^2^ scores higher than 93%. As for the MAE, it can instead be noticed that in the case of water, the algorithm reaches a higher accuracy compared to Fluid 2 and Fluid 3. In particular, the MAE returned with water is approximately 0.71 overall, with a peak of 2.66 when the model is used to predict the “warning” status. The MAE computed for the remaining fluids is greater in value, and again, it reaches a peak when trying to predict the “warning” status. The reason for the different forecasting performance of the regression model when used with the three fluids can likely be ascribed to the dependence of temperature on the rheological properties of fluids other than water, in which additives are present and affect the operating pressures. If looking instead at the different performances achieved when trying to predict the “warning” status, the difficulty could be due to the definition of that status itself, which is expected to reflect either the transition phases or a deviation (in a range between 10 and 25%) of the plant parameters from their normal condition. At the same time, however, it can also be stated that the proposed approach shows quite promising performance, as the MAE resulting from the evaluation of the system’s parameters in the “warning” status is always lower than 10% regardless of the fluid under examination.

**Figure 7:**
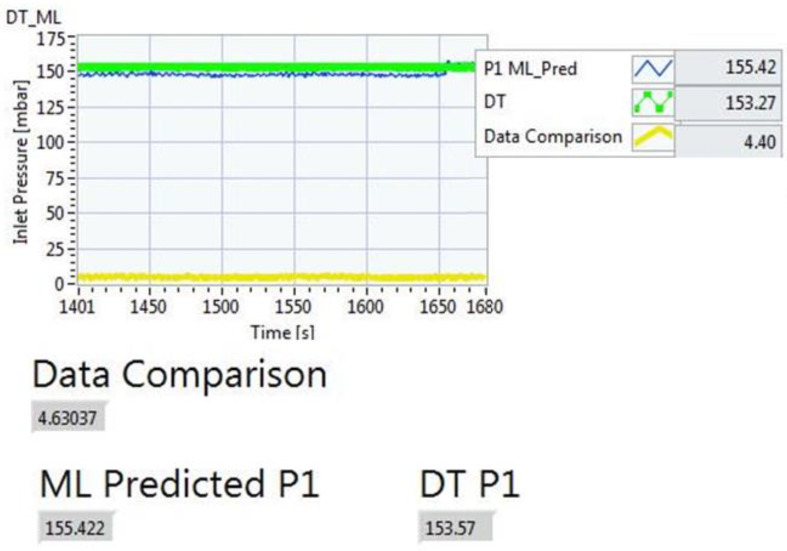
Example of online testing of the regressor model with water.

After these encouraging outcomes, the model was tested “on-line” using an ad hoc testing procedure. In particular, the pilot plant was powered up and data were acquired from the sensors in continuous mode for the three fluids considered and simulating the three possible operating conditions of “ok”, “warning”, and “failure”. The ML model was thus applied on-line using the data acquired from the pilot plant to check its capability of predicting the expected value of the outlet pressure, and if needed, to undertake actions in the plant. Figure 7 shows an example of the results that can be returned by the regressor model when used online. In particular, the picture highlights that the online usage of the regression model allows us to compute the P1 value (ML predicted P1) in real time, comparing it with the real value (Real P1) and with the estimate returned by the DT model of the plant (DT P1). The comparison between the P1 value predicted by the ML model and the real one is shown in a graph and computed analytically (“Data comparison” value).

**Figure 8:**
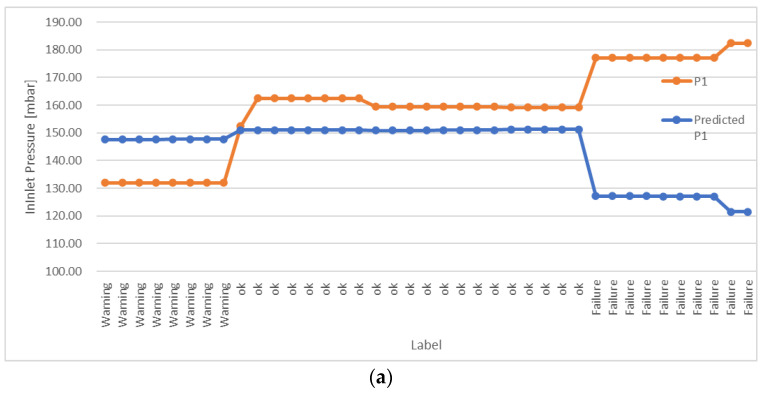

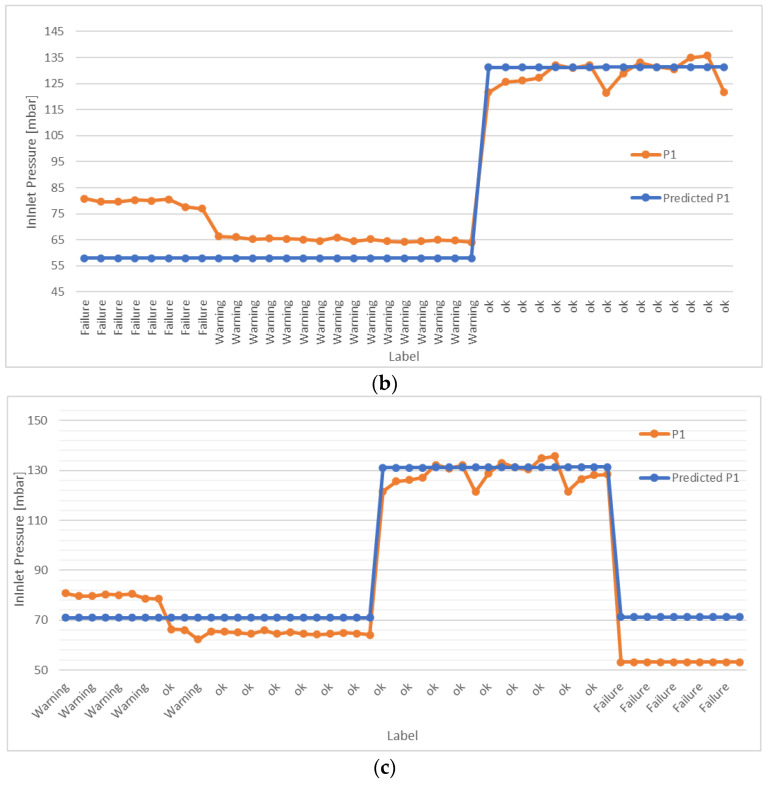
Results for the online testing of the multiple linear regression model: (**a**) Water; (**b**) Fluid 2; (**c**) Fluid 3.

The overall results obtained for the three fluids are shown in Figure 8a–c. Again, the graphs compare the real data acquired from the plant (P1 series) and those predicted using the ML algorithm (Predicted P1 series). The label on the *x*-axis indicates the machine status, reflecting its operating conditions as previously described; this was comparable with the result obtained using the DT model. As can be noticed from Figure 8, the algorithm predicts the correct inlet pressure when the machine works in the “ok” condition, meaning that it is effective in recognizing this status (error lower than 5%). For the remaining statuses, instead, the algorithm predicts the expected inlet pressure based on the outlet pressure and process flow acquired in real time. Obviously, the predicted value is different from that which is really observed, because the machine is not working in the correct way; hence, anomalous functioning can be easily detected by comparing the expected outlet pressure with that which is really observed. This result demonstrates the effectiveness of the ML implemented in the system.

### 3.2. Artificial Neural Network

The chosen artificial neural network is a Multi-Layer Perceptron Classifier (MLPC). The goal of implementing an MLPC is to return a classification of the real-time machine status on the basis of the three considered parameters. As such, the output of the algorithm is a string that describes the machine status using the three categories previously defined, i.e., “ok”, “warning”, and “failure”. The dataset for training this model is the same as that described in Section 2.3 after pre-processing; in particular, it includes the fourth column in which a label is assigned to each triplet of parameters to describe the machine status. As for the regressor algorithm, the dataset for the MPLC has been split into a training and a testing set, accounting for 70% of the dataset and for 30%, respectively. The hyperparameters set for the algorithm have been chosen following a heuristic approach. To be more precise, the number of hidden layers has been set at 2 to avoid the observed behavior of a single hidden layer’s neural network, in which nodes interact with each other and involve a worsening in the forecasting performance [24]. The number of nodes was instead varied from 50 to 1000 (step 50) in a series of preliminary tests; the results obtained in terms of forecasting accuracy and computational performance were evaluated to determine the optimal configuration, which was finally chosen for implementation.

For a neural network, a standardization of the dataset is sometimes recommended; in line with this consideration, a normalization has been applied to the dataset under evaluation, finding, as a result, that the prediction returned by the ANN is almost the same as that obtained when applying the model to the original (non-normalized) set of data, probably because of the binary orientation of the algorithm [25]. Hence, data were left in the original (non-normalized) format, so that the result returned by the algorithm is directly expressed in a meaningful measurement unit, which in turn is useful for representation purposes.

**Figure 9:**
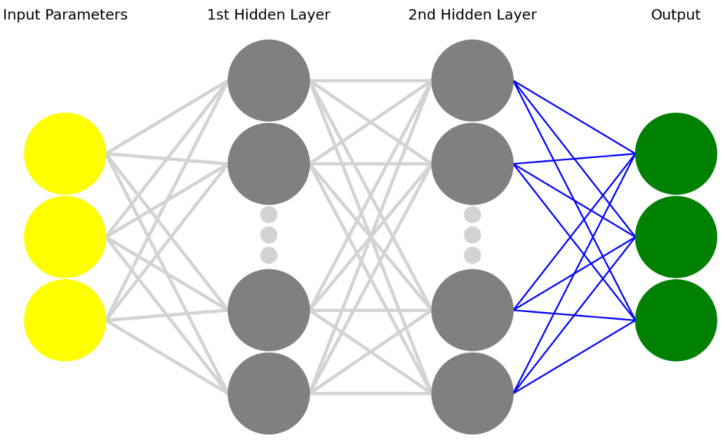
Structure of the MLPC neural network.

From a technical point of view, the final configuration of the MLPC consists of two hidden layers, in turn, composed of 200 nodes each, as shown in Figure 9. The solver adopted for optimizing the weights is the Adam optimizer, a stochastic gradient-descent procedure that updates the network weights iteratively based on the training data [26].

The validation of this ML model has been carried out via the confusion matrix [27,28], a table that displays the performance of the algorithm by evaluating its match with the real data. With this table, two main parameters can be computed, called precision and recall, using the True Positive (*TP*), False Positive (*FP*), and False Negative (*FN*) values. Because of the presence of three statuses for the machine, the case under investigation can be represented using a 3 × 3 confusion matrix, such as Table 5 below.

In this matrix, for each of the three machine statuses (denoted as *I = 1*, *…*, *3*, respectively, for ok, warning, and failure, in the equations that follow), the indexes previously mentioned can be computed using the set of formulae below:

- *TP_i_* represents the number of entries correctly predicted, meaning that their actual values are equal to the predicted one, i.e.,
  *TP_failure_* = *C*_1_ (2)
  *TP_warning_* = *C*_5_ (3)
  *TP_ok_* = *C*_9_ (4)
  *TP* = *TP_failure_* + *TP_warning_* + *TP_ok_* (5)

- *FN_i_* is the sum of the samples that were correctly predicted. Those values can be seen in each row of a category, as they represent the sum of the values listed in a row, excluding those correctly predicted (the *TP_i_* described above); the following set of formulae can thus be used for the computation:
  *FN_failure_* = *C*_2_ + *C*_3_ (6)
  *FN_warning_* = *C*_4_ + *C*_6_ (7)
  *FN_ok_* = *C*_7_ + *C*_8_ (8)
  *FN* = *FN_failure_* + *FN_warning_* + *FN_ok_* (9)

- *FP_i_* is the number of samples not belonging to class *i*, but classified as belonging to that class. Those values can be read in the column corresponding to each class, as they represent the sum of the value listed in the column excluding those correctly predicted (the *TP_i_* described above); the following set of formulae can thus be used for the computation:
  *FP_failure_* = *C*_4_ + *C*_7_ (10)
  *FP_warning_* = *C*_2_ + *C*_8_ (11)
  *FP_ok_* = *C*_3_ + *C*_6_ (12)
  *FP* = *FP_failure_* + *FP_warning_* + *FP_ok_* (13)

Using the parameters computed above, the precision (*P_i_*) of the algorithm expresses the ratio between the correct predictions over the total predictions for each category of data, while the recall (*R_i_*) denotes the ratio between the data correctly classified out of the whole set of real data. For each class, the precision is evaluated using Equations (14)–(16); Equation (17) shows instead the computation of the overall algorithm precision:

(14) $$P_{\mathrm{failure}}=\frac{TP_{failure}}{\left(TP_{failure}+FP_{failure}\right)}\times 100$$

(15) $$P_{\mathrm{warning}}=\frac{TP_{warning}}{\left(TP_{warning}+FP_{warning}\right)}\times 100$$

(16) $$P_{\mathrm{ok}}=\frac{TP_{ok}}{\left(TP_{ok}+FP_{ok}\right)}\times 100$$

*P = P_failure_ + P_warning_ + P_ok_* (17)

The average precisions (*AP_i_*) for each class can then be derived from the precision values for each class of data, weighting them by the number of instances in each class, as shown in the equations below:

(18) $${\mathrm{AP}}_{\mathrm{failure}}=\left(\frac{\sum 1r}{\sum M}\right)*P_{failure}\;$$

(19) $${\mathrm{AP}}_{\mathrm{warning}}=\left(\frac{\sum 2r}{\sum M}\right)*P_{warning}$$

(20) $${\mathrm{AP}}_{\mathrm{ok}}=\left(\frac{\sum 3r}{\sum M}\right)*P_{ok}$$

It follows that the weighted average precision (*WAP*) of the model can be obtained according to the following formula:

*WAP* = *AP_failure_* + *AP_warning_* + *AP_ok_* (21)

The model accuracy (*Acc*) is finally computed in terms of the ratio between the correctly predicted samples and the total number of samples tested; for each class, it can be computed using Equation (22):

(22) $${\mathrm{Acc}}_{i}=\left(\frac{TP_{i}}{\sum M}\right)$$

The recall values for each class (*R_i_*) are instead evaluated by applying the following equations:

(23) $$R_{\mathrm{failure}}=\frac{TP_{failure}}{\sum 1r}$$

(24) $$R_{\mathrm{warning}}=\frac{TP_{warning}}{\sum 2r}$$

(25) $$R_{\mathrm{ok}}=\frac{TP_{ok}}{\sum 3r}$$

**Figure 10:**
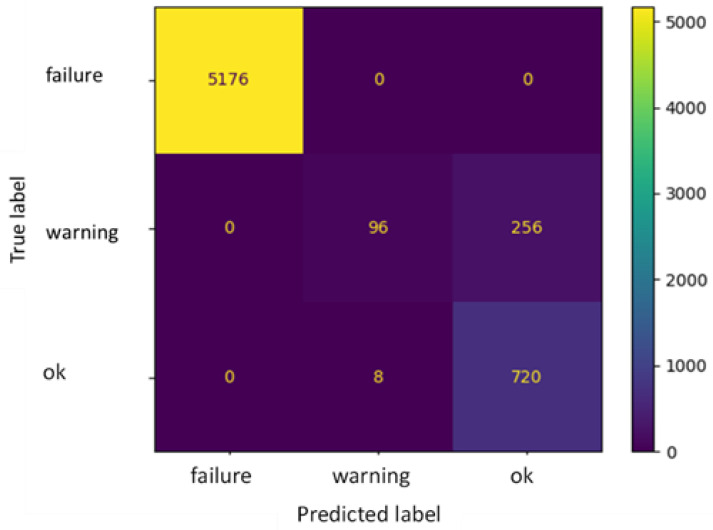
Confusion matrix for water.

Figure 10 and Table 6 show the confusion matrix and the precision values for the dataset relating to water; as can be seen from the numerical outcomes, the model accuracy reaches 96%. The MLPC model has also been evaluated using the *WAP* value, which, for the case under examination, reaches 97%.

As can be seen from Table 6, the MPLC algorithms show very good precision and recall when used for identifying the “ok” and “failure” status of the pilot plant, while the precision is significantly lower when trying to predict the “warning” status. This is in line with the results previously obtained with the linear regression model, despite its different logics compared to the MPLC. For the ML algorithm under examination, the lower forecasting performance against the “warning” status is probably due to the quite confined range of variation in the values assigned to that status (i.e., 10–25% absolute deviation from the normal condition), compared, e.g., to the wider range of the “failure” status (25–100% deviation from the normal condition). This difference clearly leads to the presence of more “failure” data in the dataset and is likely to affect the algorithm’s performance. Indeed, from Figure 10 it can also be seen that some false negative values were found for the “warning” status: as the range data for this status is limited, the ML algorithm needs to be very precise to accurately detect them.

**Figure 11:**
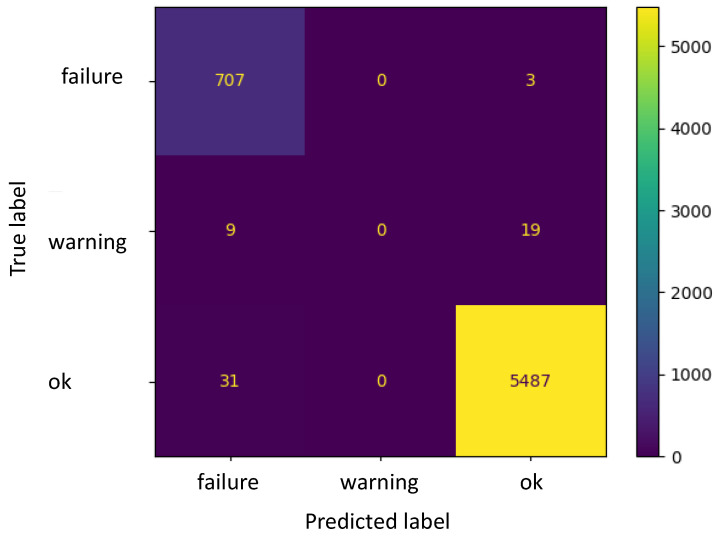
Confusion matrix for Fluid 2.

**Figure 12:**
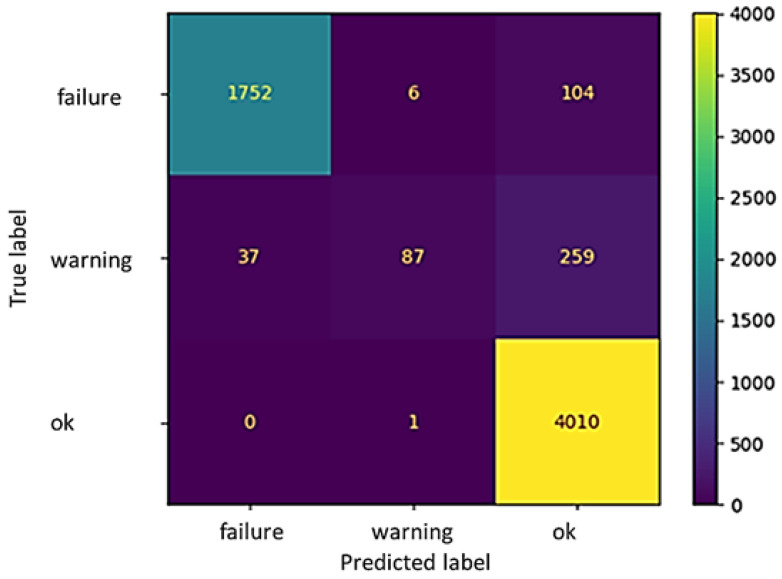
Confusion matrix for Fluid 3.

As for the previous result, MLPC exhibits a high accuracy (approximately 99%) for Fluid 2 (Figure 11 and Table 7), and an accuracy of 93% for Fluid 3 (Figure 12 and Table 8). As far as the *WAP* is concerned, the recall value scores zero for the “warning” status of Fluid 2 due to the absence of data classified in this category. For Fluid 3, some false negative classifications are observed when evaluating the “failure” status: out of the total number of failure situations, approx. 5% were instead classified as “ok”. Under these circumstances, if using the proposed algorithm, the machine would continue to work, while in reality, it should have been stopped. Implications of these situations will be discussed in the conclusions.

### 3.3. K-Means Clustering

Clustering is an unsupervised ML algorithm that involves dividing an entire dataset into groups (i.e., clusters) based on some common characteristics. The k-means algorithm, in particular, groups a set of samples into separate clusters described by the mean value (centroid) of the elements in the cluster. In k-means clustering, the similarity between elements is therefore expressed in terms of its closeness to the centroid of the clusters. As for the previously described algorithms, the dataset on which the clustering algorithm was applied consists of the three columns that list the key parameters of the plant under examination (i.e., P1, P2, and F). From a technical point of view, a clustering algorithm can sometimes experience problems if used in datasets with outliers or noisy data. However, this is not the case for our implementation context. Indeed, the dataset consists of triplets of data that were always collected in controlled conditions, in line with the obvious need for avoiding safety problems for the operators or corrupted machine functioning. It is very unlikely that the testing environment will generate outliers.

In line with our previous consideration about the usage of the clustering algorithm (cf. Section 2.4), the expected outcome of this approach is the classification of the triplets of data collected into the appropriate status of “ok”, “warning”, or “failure”. This implicitly means that the algorithm is expected to classify the elements in the dataset into three clusters, corresponding to the three statuses of functioning. However, the number of clusters in the k-means procedure is not determined a priori; rather, the first step in applying this algorithm consists of determining the optimal number of clusters (denoted as k) for the dataset. This parameter, as well as other hyperparameters of the algorithm, has been set following a heuristic approach. To be more precise, the number of clusters has been determined via the elbow method; this consists in plotting the explained variation as a function of the chosen number of clusters, and then taking the elbow (“knee”) of the curve as the optimal number of clusters to use [29,30]. Accordingly, the sum of squared error (SSE) between the data points and their assigned clusters’ centroids was evaluated whilst varying the number of clusters from 1 to 6 (step 1). The initial clusters’ composition was determined by assigning each item to a random cluster. Then, the algorithm follows an iterative procedure, by which the distance of the clusters’ elements from the centroid is evaluated and the elements that are found to be too distant are moved to a different cluster. The goal of this procedure, which consists of a maximum of 15 iterations, is to ensure that the elements assigned to groups are characterized by a high degree of similarity, whereas clusters must be relatively distant from each other.

**Figure 13:**
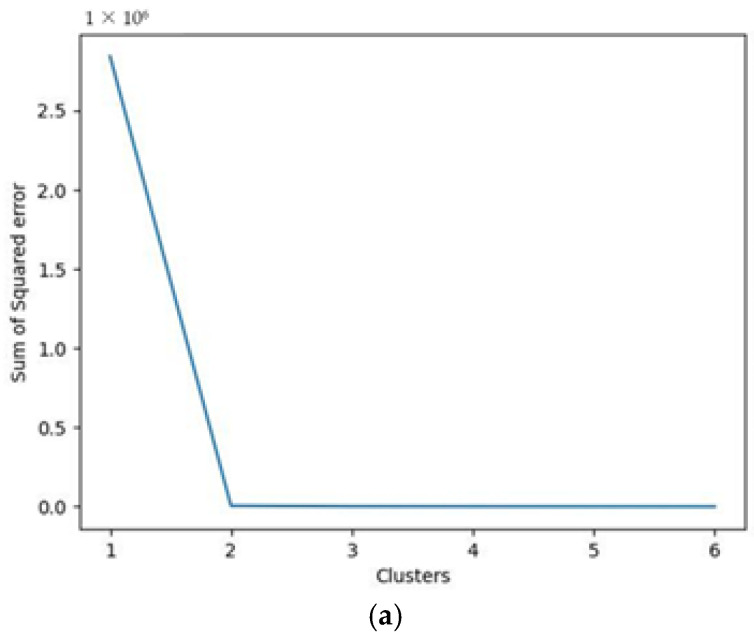

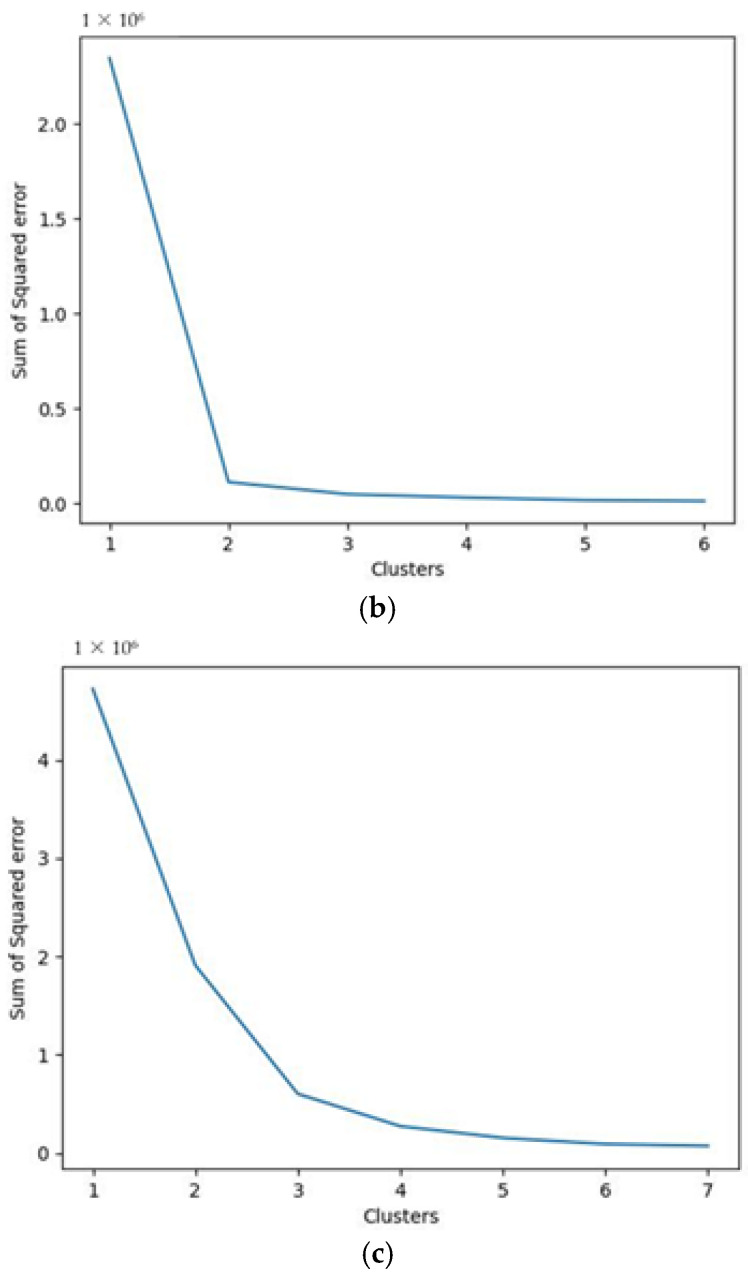
Elbow plot for the three datasets: water (**a**), Fluid 2 (**b**), and Fluid 3 (**c**).

The graphs in Figure 13a–c show the elbow curve obtained for the dataset under examination (water, Fluid 2, and Fluid 3, respectively). As can be seen from the outcomes obtained, for water and Fluid 2, it would be reasonable to set the number of clusters at three, as with this value, the trend of both curves reaches a sufficient stability of the variance explained. On the contrary, the same consideration does not hold true for Fluid 3, for which, instead, stability in the SSE is reached with a greater number of clusters (six). Nonetheless, to confirm the suitability of using the clustering algorithm in the system under examination, the number of clusters was set to three for all fluids.

**Figure 14:**
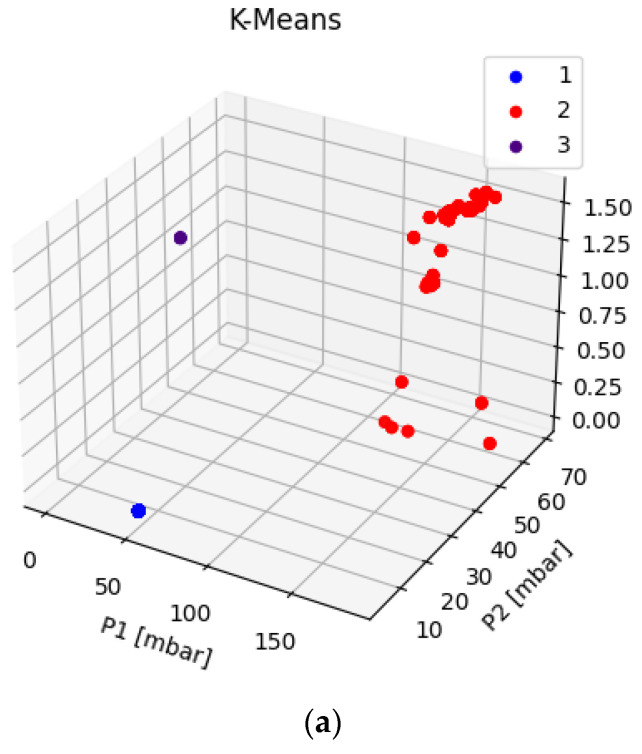

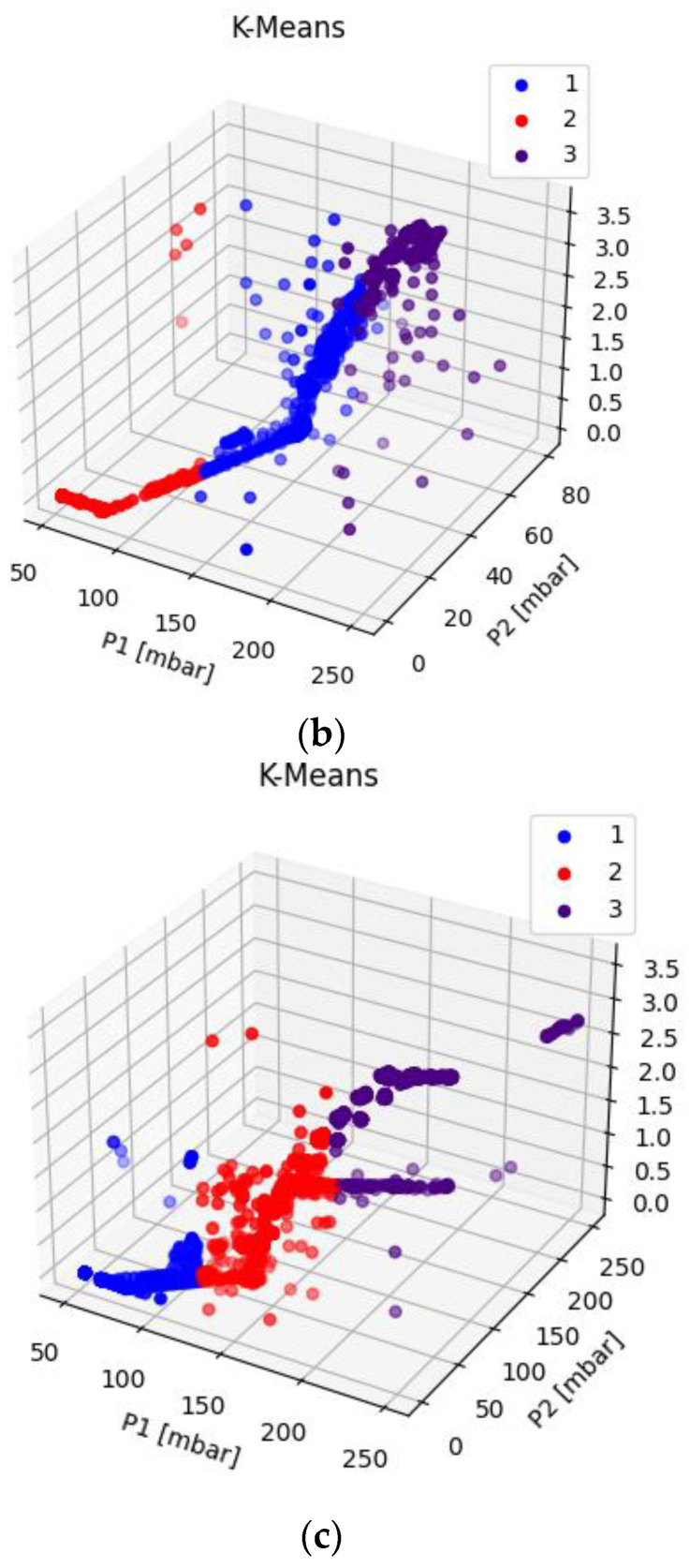
k-means plots for water (**a**), Fluid 2 (**b**), and Fluid 3 (**c**).

With this number of clusters, the k-means algorithm was used to classify the elements of the dataset to check its capability of identifying the “ok”, “warning”, and “failure” status of the system. A 3D plot of the clusters obtained for the three fluids is shown in Figure 14a–c; the classification returned by the algorithm was also saved in a new column of the database, simply called a cluster, which was filled with the value predicted by the k-means model.

The silhouette score [30] was used as a performance index to evaluate the correctness of the classification. The technique provides a synthetic representation of how well each element has been classified, and therefore evaluates the accuracy of a clustering technique. The score ranges from −1 to 1, where:

- A score of 1 means clusters are clearly distinguished;
- A score of 0 means the distance between clusters is not significant;
- A score of −1 means clusters are assigned in the wrong way.

The models developed had a silhouette score of 0.92 for water, 0.91 for Fluid 2, and 0.76 for Fluid 3, which suggests that, even if forcing the number of clusters to be three, the algorithm is able to correctly classify them. The fact that the clusters are well-defined, however, does not imply that they correctly capture the possible machine status, as the clustering algorithm would never be able to label the clusters as “ok”, “warning”, or “failure”. To check this aspect, an analysis was conducted to compare the original status of the machine with the three clusters obtained after the classification. The results are shown in Table 8, Table 9 and Table 10.

As shown in Table 9, the clusters identified by the k-means algorithm for the dataset of water can be summarized as follows. Clusters 1 and 3 only include “failure” data, with different characteristics. To be more precise, “failure” situations of cluster 3 refer to triplets of data in which the inlet pressure is always null, while “failure” situations in cluster 1 are characterized by almost no flow of product. No matter the specific scenario, however, these failure situations appear to be correctly detected by the algorithm. Instead, cluster 2 includes a mixture of “ok” and “warning” statuses, with a residual quota of “failure” situations. This means that the clustering algorithm is unable to discriminate between the “ok” and “warning” situations, which, once again, denotes a difficulty in detecting the “warning” status of the machine, as already observed for the remaining ML algorithms tested. Similarly, the composition of clusters for Fluid 2 (Table 10) shows that one cluster (i.e., cluster 2) is very well-defined and includes only normal working conditions of the plant, while clusters 1 and 3 include a mixture of possible situations. Cluster 3 could be somewhat acceptable in practice, as the majority of the data refer to the “ok” status. The classification returned by cluster 1 is not sufficiently precise for practical usage, however, as 24% of the anomalous situations are assimilated to normal working conditions of the plant, which could be dangerous for an employee working on the machine. As far as Fluid 3 is concerned (Table 11), although the algorithm was forced to use three clusters instead of the ideal number of six, the clusters seem to correctly capture most of the real working conditions of the plant. To be more precise, cluster 1 only consists of “failure” situations, and similarly, cluster 3 almost only contains “failure” situations. This outcome suggests that anomalous machine functioning was again correctly detected and classified by the algorithm. Looking instead at cluster 2, we can see that its composition includes all the “ok” statuses, with small quotas of warning and failure situations. Once again, the “warning” status, thus, appears to be somehow assimilated into the “ok” or “failure” condition.

## 4. Discussion and Conclusions

This work has proposed an application whose aim is to integrate digital twin models, machine-learning algorithms, and Industry 4.0 technologies, to design a comprehensive tool for detecting anomalies in the functioning of an industrial system. The proposed solution has been designed to be suitable for implementation in a tube-in-tube indirect machine for fluid food pasteurization. To achieve good results with the available technologies, four different modes of functioning have been developed and implemented in the digital twin of the plant. The series of tests carried out aimed at demonstrating which operating mode fits best the real condition of the system.

The DT environment, via the tools developed in previous studies, can be used for monitoring and controlling the system, both in situ and via remote connection. However, the need to manually adjust the setpoint of the controller and set the fluid characteristics in the software can represent a limitation compared to the fully automated functioning of the tool. To overcome this issue, three ML approaches (namely, a linear regression model, an artificial neural network, and a clustering algorithm) have been embodied in the solution developed and implemented for the online monitoring of the plant. In this respect, this study was exploratory in nature, and aimed to test various ML algorithms in an attempt to find the most effective one for anomaly detection; in this field of application, research on ML adoption is still limited. The results obtained using these ML tools showed that the regression algorithm, once integrated into a DT environment, could be a suitable way to obtain automatic control of the system. Indeed, the dependent variable predicted by the algorithm (P1) on the basis of multiple independent variables (P2 and F) and returned as an output in the form of a discrete value, can directly be used as a setpoint for the PID. At the same time, the DT model operates as a mirror of the machine behavior, supervises system functioning, and can even stop the machine from functioning in case a “failure” is detected, by displaying the machine status on the HMI. The artificial neural network and clustering algorithms, instead, returned slightly worse performances. In particular, the MLPC algorithm had a high accuracy as an anomaly prediction tool when used for classifying the “ok” or “failure” status of the different fluids tested, while a lower precision was reached in the classification of the “warning” status. Similar conclusions can be made for the k-means clustering algorithm, which is generally able to group the “failure” and “ok” status, while the “warning” status is often confused with the correct functioning of the plant. This outcome can be justified based on the definition of the “warning” status itself, which includes situations in a quite limited range of values, and therefore, it is difficult to precisely detect them. At the same time, the “failure” status is not always correctly captured by the artificial neural network and clustering algorithms tested. This is a current limitation of the proposed approaches and deserves attention in future studies. False-negative classifications of the “failure” status have to be avoided, if possible, as they denote situations in which, if using the classification returned by the MPLC and k-means algorithms, the machine would continue to work, while it actually needs to be stopped. Although these false-negative values are quite few in number, they are also the most dangerous for the employee safety; therefore, for being used to the “failure” status of the machine, the MPLC and k-means algorithms need some preliminary refinements. Otherwise, in practical cases, it could be advisable to use both approaches and couple their results; indeed, the possibility that both methods return false negative values for the “failure” status is obviously lower than that of each method being used singly.

In view of an in-field implementation, therefore, the usage of the clustering or classification algorithms for anomaly detection would need to be limited to the identification of the “ok” situations, which are typically correctly captured. This means that anytime the operating conditions deviate from this status, a message should be displayed on the HMI to alert an employee about a possible need for intervention at the plant. In addition, there are some further aspects that need to be addressed in practical cases. One of these aspects is that a clustering algorithm can return bad performance values if used on a dataset that includes outliers or noisy data. Although this is not the case for our study, this circumstance could always be observed in real cases, and would involve some preliminary activities and checks to be made on the data collected.

## Figures and Tables

**Table 1:** Sensor model, ID, and process parameters.

Sensor Model	ID	Process Parameters	Range
S11-Wika	P1	Inlet Pressure	0–250 (mbar) relative Pressure
S11-WIka	P2	Outlet Pressure	0–250 (mbar) relative Pressure
Optimass–Krohne	F	Product Flow	0–5 (m^3^/h)

**Table 2:** Parameters’ variation for each run of data collection.

Parameter	Run 1	Run 2	Run 3
Operating Temperature (°C)	20	40	60
Inverter Range (%)	0–100%	0–100%	0–100%
Sample Rate (Hz)	1	1	1

**Table 3:** Summary of the data collected.

	Count	Mean	Std	Min	25%	50%	75%	Max
P1 Water	6256	70.30	40.7	0	50.01	50.02	50.03	182.38
P1 Fluid 2	6256	145.68	41.52	52.9	137.65	151.45	169.2	250
P1 Fluid 3	6256	154.8	47.21	49.07	141.14	162.27	185.9	250
P2 Water	6256	14.08	21.31	3.27	3.36	3.40	3.42	68.54
P2 Fluid 2	6256	47.61	19.32	0.06	53.87	55.79	57.24	80.52
P2 Fluid 3	6256	51.87	27.33	0.49	53.81	56.78	58.82	250
F Water	6256	0.29	0.58	0.007	0.008	0.009	0.011	1.55
F Fluid 2	6256	1.49	1.11	0	0.69	1.48	2.33	3.62
F Fluid 3	6256	1.51	1.11	0	0.56	1.53	2.33	3.53

**Table 4:** Linear regression multiple variable results.

	*a*_1_	*a*_2_	*Intercept*	MAE	R^2^
Water (fluid 1)	1.65	8.98	44.32	0.71	0.99
Fluid 2	1.27	18.29	57.28	5.745	0.947
Fluid 3	0.77	28.87	70.65	7.467	0.938

**Table 5:** 3 × 3 confusion matrix.

		Predicted			
		Failure	Warning	Ok	
True	Failure	*C*_1_ *(TP_failure_)*	*C*_2_	*C*_3_	$\sum 1r=C_{1}+C_{2}+C_{3}$
	Warning	*C*_4_	*C*_5_ *(TP_warning_)*	*C*_6_	$\sum 2r=C_{4}+C_{5}+C_{6}$
	ok	*C*_7_	*C*_8_	*C*_9_ *(TP_ok_)*	$\sum 3r=C_{7}+C_{8}+C_{9}$
		$\sum 1c=C_{1}+C_{4}+C_{7}$	$\sum 2c=C_{2}+C_{5}+C_{8}$	$\sum 3r=C_{3}+C_{6}+C_{9}$	*TP* = *TP_failure_* *+ TP_warning_ + TP_ok_*

**Table 6:** Average performance values of the ANN model for water.

Category	Precision (*P_i_*)	Recall (*R_i_*)	Average Precision (*AP_i_*)
Failure	1.00	1.00	0.83
Warning	0.92	0.27	0.05
Ok	0.74	0.99	0.09
Weighted average precision (*WAP*)			0.97
Model accuracy (*Acc*)			0.96

**Table 7:** Average performance values of the ANN model for Fluid 2.

Category	Precision (*P_i_*)	Recall (*R_i_*)	Average Precision (*AP_i_*)
Failure	0.95	1.00	0.11
Warning	-	-	-
Ok	1.00	0.99	0.88
Weighted average precision (*WAP*)			0.99
Model accuracy (*Acc*)			0.99

**Table 8:** Average performance values of the ANN model for Fluid 3.

Category	Precision (*P_i_*)	Recall (*R_i_*)	Average Precision (*AP_i_*)
Failure	0.98	0.94	0.29
Warning	0.93	0.23	0.06
Ok	0.92	1.00	0.59
Weighted average precision (*WAP*)			0.94
Model accuracy (*Acc*)			0.93

**Table 9:** Cluster composition vs. machine status for water.

	Cluster Composition		
Machine status	1	2	3
OK	-	58%	-
Warning	-	28%	-
Failure	100%	14%	100%

**Table 10:** Cluster composition vs. machine status for Fluid 2.

	Cluster Composition		
Machine status	1	2	3
OK	76%	100%	87%
Warning	-	-	1%
Failure	24%	-	12%

**Table 11:** Cluster composition vs. machine status for Fluid 3.

	Cluster Composition		
Machine status	1	2	3
OK	0%	83%	0%
Warning	-	7%	1%
Failure	100%	10%	99%

## Data Availability

Data for this study are available as Supplementary Materials and include the dataset used for the training and testing of the ML algorithms (MS Excel^TM^) and a video of the online usage of the linear regression model.

## Supplementary Materials

The following supporting information can be downloaded at: https://www.mdpi.com/article/10.3390/s22114143/s1, Table S1: dataset used for training and testing of the ML algorithms; Video S1: online testing of the regression model.
